# Analysis of the interrelationships between factors influencing blood pressure in adults

**DOI:** 10.11606/s1518-8787.2020054002123

**Published:** 2020-12-04

**Authors:** Rafael Silveira Freire, Vivianne Margareth Chaves Pereira Reis, Alexandre Botelho Brito, Maria Fernanda Santos Figueiredo Brito, Lucinéia de Pinho, Rosângela Ramos Veloso Silva, Marise Fagundes Silveira

**Affiliations:** I Faculdades Unidas do Norte de Minas Instituto de Ciências da Saúde Montes ClarosMG Brasil Faculdades Unidas do Norte de Minas . Instituto de Ciências da Saúde . Montes Claros , MG , Brasil; II Universidade Estadual de Montes Claros Departamento de Educação Física Montes ClarosMG Brasil Universidade Estadual de Montes Claros . Departamento de Educação Física . Montes Claros , MG , Brasil; III Instituto Federal do Norte de Minas Gerais Departamento de Matemática Montes ClarosMG Brasil Instituto Federal do Norte de Minas Gerais . Departamento de Matemática . Montes Claros , MG , Brasil; IV Universidade Estadual de Montes Claros Programa de Pós-Graduação em Cuidado Primário em Saúde Montes ClarosMG Brasil Universidade Estadual de Montes Claros . Programa de Pós-Graduação em Cuidado Primário em Saúde . Montes Claros , MG , Brasil; V Universidade Estadual de Montes Claros Programa de Pós-Graduação em Ciências da Saúde Montes ClarosMG Brasil Universidade Estadual de Montes Claros . Programa de Pós-Graduação em Ciências da Saúde . Montes Claros , MG , Brasil

**Keywords:** Adult, Middle Aged, Hypertension, epidemiology, Health Behavior, Risk Factors, Socioeconomic Factors, Cross-Sectional Studies

## Abstract

**OBJECTIVE:**

To investigate the interrelationships between sociodemographic, behavioral and clinical factors associated with high blood pressure in a population of Brazilian adults.

**METHODS:**

Data from a cross-sectional population-based study conducted with adults were used. In the hypothetical model developed socioeconomic status, fruit and vegetable intake, adiposity and blood pressure were treated as latent variables and age, gender, glycemia, physical activity, smoking, alcohol consumption and control of arterial hypertension were considered observed variables. Confirmatory factorial analysis was used to construct the latent variables measurement models and the structural equation modeling was used to adjust the final model.

**RESULTS:**

The study included 808 individuals, with mean age of 44.2 years (± 17.8), 52.7% being female. It verified that age exerted a positive direct effect on blood pressure (β = 0.39), adiposity (β = 0.44), glycemia (β = 0.26) and smoking (β = 0.30). Age had a negative direct effect on physical activity (β=-0.17) and alcohol consumption (β = -0.10). Males were positively associated with blood pressure (β = 0.13), smoking (β = 0.28; p < 0.001) and alcohol consumption (β = 0.18). Adiposity had a positive direct effect on blood pressure (β = 0.23) and glycemia (β = 0.16) and alcohol consumption produced a positive effect (β = 0.09) on adiposity. Fruit and vegetable intake had a negative direct effect on blood pressure (β = -0.11), while socioeconomic status had a positive direct effect on fruit and vegetable consumption (β = 0.47). We adjusted the structural model according to the variable medical control of arterial hypertension, which had a negative direct effect on blood pressure (β = -0.10).

**CONCLUSIONS:**

Results suggest that increasing age is associated with increased blood pressure, adiposity, glycemia and smoking, as well as with reduced physical activity and alcohol consumption. Males were associated with increased blood pressure and greater use of alcohol and cigarettes. Higher adiposity indicators were correlated with increased blood pressure and glycemic levels; higher alcohol consumption was associated with increased adiposity. Higher consumption of fruits and vegetables, as well as active control of hypertension were associated with reduced blood pressure. Better socioeconomic status was associated with higher consumption of fruits and vegetables.

## INTRODUCTION

Cardiovascular diseases (CVD) are the main causes of morbidity and mortality worldwide, responsible for nearly 17 million deaths per year. Of these, approximately 55% are complications resulting from systemic arterial hypertension (SAH), constituting one of the ten leading causes of death in the world ^1^ .

The overall prevalence of SAH in adults was estimated at 22%, with higher prevalence in regions of Africa (30%), while in the Americas, Europe, Asia and Oceania, the prevalence ranged from 18% to 25% ^2^ . In Brazil, SAH is considered one of the most relevant public health problems, with a prevalence of approximately 30% in adults, reaching more than 50% between 60 and 69 years and 75% over 70 years ^3^ .

Given its significant prevalence and mortality rate in the global population, a significant number of studies in the literature seek to estimate the effects of factors associated with SAH, including genetic, environmental and behavioral factors ^4^ . However, this issue has been more commonly analyzed using classical statistical techniques, known for contemplating multiple risk factors, but limited to a single relationship between them and the outcome.

On the other hand, high blood pressure (BP) is a phenomenon influenced by a set of interrelated factors ^3^ . Some of these factors, such as obesity and diabetes mellitus, act directly, while others act indirectly via intermediate factors, including habits and behaviors such as excessive alcohol consumption, smoking, physical inactivity or poor nutrition. The more distal risk factors, such as schooling and income, have less evident direct associations. However, changes in intermediate or distal factors may influence proximal risk factors and result in greater effect size on high BP. Therefore, there is a complex network of interrelated factors that influence blood pressure levels and that can be analyzed simultaneously to better understand this phenomenon and the existing relationships between variables. However, no other studies have been identified that investigated these interrelations using structural equation modeling.

Given this context, this study aimed to investigate the interrelationships between sociodemographic, behavioral and clinical factors associated with high BP in a population of Brazilian adults.

## METHODS

We used data from the study titled “Leptin receptor gene dfn223arg polymorphism is not associated with hypertension: a preliminary population-based cross-sectional study” ^7^ . This is an epidemiological, cross-sectional, population-based study conducted with individuals aged 18 years or older, residing in the urban region of the city of Montes Claros, Minas Gerais, Brazil, in 2013.

Sample size calculation, as described by Pena et al. ^7^ , estimated the participation of at least 750 adults. To this end, they considered expected frequency of 10% leptin receptor polymorphism, coefficient of variation of less than 25%, standard error less than 3% and correction for the design effect ( *deff* ) of 2.0 ^6^ . The sample size set for the survey, thus, meet the assumptions for the sample size in the present study, which aiming to estimate a prevalence of hypertension of 30% ^2 , 4^ , with confidence level of 95%, margin of absolute error of 5%, correction for the design effect of 2.0 and a 10% increase in the rate of non-responders, estimated a minimum of 710 participants.

Probabilistic conglomerate sampling was adopted in two stages. In the first, census tracts were drawn, and in the second, households. Prior to data collection, researches underwent training and calibration, and a pilot study with a convenience sample was conducted.

Data collection took place in the selected households, via a structured questionnaire consisting of variables related to sociodemographic characteristics and health-related behaviors. We also measured anthropometric variables, BP and capillary dfycemia ^7^ .

Sociodemographic characteristics included gender, age, schooling, socioeconomic class and family income. Schooling level was assessed according to the last grade completed by the individual and classified into 10 ordinal categories (1: illiterate; 2: incomplete 4th grade; 3: complete 4th grade; 4: incomplete 8th grade; 5: Complete 8th grade; 6: incomplete high school; 7: complete high school; 8: incomplete undergraduate studies; 9: complete undergraduate studies and 10: postgraduate). Socioeconomic class was assessed by the Brazilian Economic Classification Criteria, whose scale varies from 0 to 46 points, where the higher the score, the better the socioeconomic classification of individuals ^8^ . Family income was expressed by minimum wages (MW), with the current salary of R$ 622.00, and divided into six ordinal categories (1: < 1 MW; 2: 1 to 1.99 MW; 3: 2 to 3.99 MW; 4: 4 to 5.99 MW; 5: 6 to 7.99 MW; and 6: ≥ 8 MW).

Health-related behaviors were assessed by the variables physical activity (PA), fruit and vegetable intake, alcohol consumption and smoking. PA was measured using the International Physical Activity Questionnaire (IPAQ), long form ^9^ . We calculated the weekly time (in minutes) of walking and moderate and vigorous physical activity in the domains of work, means of transportation, home (domestic activities) and recreation, sports, exercise and leisure. Fruit and vegetable consumption was assessed by the weekly frequency consumption of raw vegetables, salad, cooked vegetables, natural juice and fruit. The categorization of these variables was expressed by the following ordinal scale: 0: never/hardly ever; 1: one to two days a week; 2: three to four days a week; 3: five to six days a week and 4: every day of the week. Alcohol consumption was calculated by multiplying the weekly frequency by the number of doses ingested on each occasion. Smoking was measured in packs-year, which represents the product of the number of packs smoked per day (1 pack = 20 cigarettes) by the number of years as an active smoker.

Anthropometric measurements were taken in duplicate and included weight (in kilograms), height (in meters) and waist circumference (WC) (in centimeters). The techniques used to obtain these measures followed the World Health Organization (WHO) protocols ^10^ . Body weight was measured using a portable scale (model PL 150, GTech) accurate to 0.1 kg. Height was measured by a portable stadiometer (Alturexata) with metal base and height leveling apparatus acting as the square accurate to 0.1 cm. Waist circumference was measured by a non-stretchable tape measure (TBW) accurate to 1 mm. Body mass index (BMI) was calculated according to the formula: BMI = weight (kg) / height ^2^ (m). We also calculated the waist-to-height ratio (WHR), dividing WC by height, both measured in centimeters.

BP was measured using a calibrated digital sphygmomanometer (model HEM-7200, Omron), according to the protocol of the Brazilian Guidelines for Arterial Hypertension ^11^ , with three measurements being performed at a five-minute interval. In addition, we collect information about medical control of arterial hypertension, by the following questions: “Has the doctor ever diagnosed you with hypertension or high blood pressure? (yes or no)” and “If yes, do you take medication to control it? (yes or no).” Capillary glycemia was measured by capillary puncture, using a digital glucometer (model Accu-Check ^®^ Performa, Roche Diagnostics).

Socioeconomic status, fruit and vegetable intake, adiposity and BP were considered latent variables. Socioeconomic status was defined by three observed variables: schooling, socioeconomic class and family income. Fruit and vegetable consumption comprised the variables: consumption of raw vegetables, salad, cooked vegetables, natural juice and fruit. Adiposity was measured by the variables BMI, WC and WHR in the first measurement and BP by systolic blood pressure (SBP) and diastolic blood pressure (DBP) in all three measurements.

**Figure 1:**
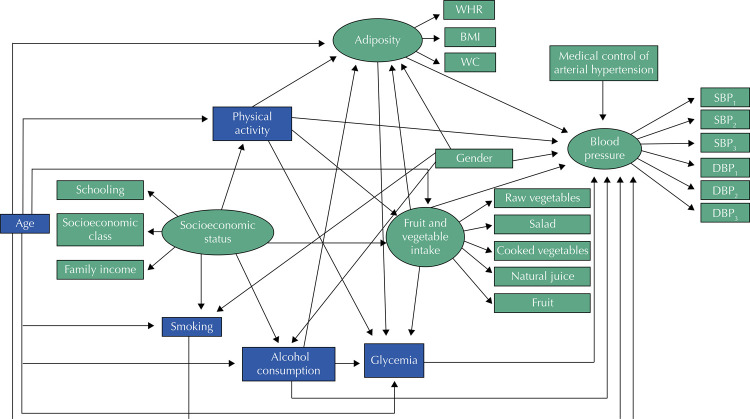
Hypothetical model tested to evaluate the interrelations between factors influencing blood pressure in adults (≥ 18 years). Montes Claros, Minas Gerais, 2012-2013 (n = 808).

WHR: waist-to-height ratio; BMI: body mass index; WC: waist circumference; SBP: systolic blood pressure; DBP: diastolic blood pressure.

We developed a multivariate theoretical model, adapted from the conceptual model of risk factors for coronary artery disease. We sought to evaluate the interrelationships between BP, considered as the main outcome, and the other variables: gender, age, socioeconomic condition, fruit and vegetable consumption, PA, alcohol consumption, smoking, adiposity, capillary glycemia and medical control of arterial hypertension. Figure 1 illustrates the direct and indirect relationships between the variables investigated in the proposed model. The observed variables are represented by rectangles, the constructs by ellipses, and the correlations by arrows (from the independent variable to the dependent) ^12^ .

In the data analysis, categorical variables were described by their frequency distributions; and numerical variables by mean, standard deviation (SD), minimum and maximum values, skewness coefficient (sk) and kurtosis (ku), with correction by the design effect ( *deff* ), as they come from a cluster sampling. To incorporate the structure of the complex sampling plan in the descriptive analysis of the variables and correct the loss of accuracy in the estimates, each respondent was associated with a weight inversely related to their probability of inclusion in the sample. Values of sk > 3 and ku > 10 were considered as indicators of normality assumption violation ^12^ . The variables alcohol consumption, PA, smoking and capillary glycemia received logarithmic transformation because they violated the normality assumption. Lost values were imputed by the mean.

Model adjustment was carried out in two stages. First, we adjusted the measurement models, which established how latent variables were measured. To this end, confirmatory factor analysis (CFA) was used. Latent variables that presented all significant factor weights (p < 0.05) and standardized factor weights greater than or equal to 0.50 (λ ≥ 0.50) were considered well-adjusted ^12^ .

Subsequently, the multivariate model was adjusted by structural equation modeling (SEM). Direct and indirect effects were estimated, represented by standardized coefficients, whose statistical significance was assessed by critical ratio (CR), at a 5% level. The standardized structural coefficients express the variation, in standard deviation, in the dependent variable by one-unit variation of the standard deviation in the independent variable. When positive, they indicate that the variables “walk” in the same direction, that is, the increase in the independent variable is correlated with the increase in the dependent variable. On the other hand, when the coefficient is negative, it indicates that the increase in the independent variable is correlated with a reduction in the values of the independent variable. Standardized coefficients with values close to 0.10, 0.30 and greater than 0.50 were interpreted as small, medium and large effects, respectively ^12^ .

For evaluating the fit quality of the models, we used the Bentler’s comparative fit index (CFI), goodness-of-fit index (GFI) and Tucker-Lewis index (TLI), considering values greater than 0.90 as indicative of a good fit ^13^ . We also used the root mean square error of approximation (RMSEA), whose values lower than 0.10 were considered indicators of reasonable fit. The absolute index X ^2^ /df was also adopted, considering a value lower than 5 as indicator of an acceptable fit ^13^ .

For descriptive analyses, we used the *complex sample* module implemented in IBM SPSS 23.0 software. The measurement and structural models were adjusted using IBM SPSS Amos 23.0 software and R 3.5.0 software, respectively.

All participants signed the Informed Consent Form. The research project that originated this study was approved by the research ethics committees of Universidade Estadual de Montes Claros (opinion No. 226,604/2013) and Universidade Federal de Minas Gerais (opinion No. 213,555/2013).

## RESULTS

The study comprised 808 individuals, with mean age of 44.2 years (SD = 17.8), of which 52.7% were female. Monthly family income was less than two minimum wages for approximately half (46%) of the interviewees, and 39.5% had less than high school schooling. Mean SBP was 129.5 mmHg (SD = 21.3) and mean DBP 76.1 mmHg (SD = 12.0). Of the interviewees, 26.3% had high BP (SBP ≥ 140 mmHg and/or DBP ≥ 90 mmHg) and 17.7% reported medically controlling arterial hypertension. Table 1 shows the descriptive measures of the other characteristics of the participants.

**Table 1:** Variables related to sociodemographic conditions, behavioral and clinical aspects in adults (≥ 18 years). Montes Claros, Minas Gerais, 2012-2013.

Variable	n	% ^a^	Variable		n	% ^a^
**Schooling level (n = 806)**			**Salad intake (n = 808)**			
Illiterate	62	5.5	Never/hardly ever		28	3.7
Incomplete 4th grade	99	9.7	1 to 2 days/week		85	11.7
Complete 4th grade	101	10.8	3 to 4 days/week		194	25.5
Incomplete 8th grade	54	7.1	5 to 6 days/week		158	18.9
Complete 8th grade	56	6.4	Every day		343	40.2
Incomplete high school	60	9.3	**Cooked vegetables intake (n = 797)**			
Complete high school	260	36.3	Never/hardly ever		89	10
Incomplete undergraduate schooling	58	8.4	1 to 2 days/week		249	31.6
Complete undergraduate schooling	48	5.6	3 to 4 days/week		220	29.3
Graduate	9	0.9	5 to 6 days/week		130	15.9
**Income in MW (n = 807)**			Every day		109	12.1
Less than 1	23	3.4	**Natural juice consumption (n = 808)**			
From 1 to 1.99	347	42.6	Never/hardly ever		211	28.2
From 2 to 3.99	348	43.2	1 to 2 days/week		237	28.4
From 4 to 5.99	46	5.9	3 to 4 days/week		188	24.2
From 6 to 7.99	23	3.0	5 to 6 days/week		95	10.5
8 or more	20	1.9	Every day		77	8.8
**Raw vegetable intake (n = 794)**			**Fruit intake (n = 808)**			
Never/hardly ever	92	10.9	Never/hardly ever		69	8.7
1 to 2 days/week	243	31.0	1 to 2 days/week		189	26.6
3 to 4 days/week	193	25.4	3 to 4 days/week		203	24.2
5 to 6 days/week	124	15.2	5 to 6 days/week		133	15.4
Every day	142	17.4	Every day		214	25.1

**Variables**	**Mean (SD) ^a^**		**Min**	**Max**	**sk**	**ku**

Age	44.2 (17.8)		18.0	91.0	0.44	-0.61
ECC score	18.0 (5.8)		6.0	42.0	1.03	1.40
SBP ^b^	129.5 (21.3)		86.7	209.3	0.88	0.77
DBP ^b^	76.1 (12.0)		46.7	122.0	0.50	0.30
WHR	0.54 (0.09)		0.36	0.87	0.40	0.09
BMI	26.2 (5.6)		14.9	53.8	0.96	1.73
WC	87.3 (13.7)		58.0	143.0	0.47	0.33
Capillary glycemia	121.7 (53.9)		58.0	600.0	2.28 ^c^	7.68 ^c^
Physical activity	800.5 (834.0)		0.0	1.960	1.52 ^c^	2.08 ^c^
Alcohol consumption	4.3 (9.8)		0.0	41.0	1.90 ^c^	2.48 ^c^
Smoking	4.8 (13.8)		0.0	120.0	1.89 ^c^	2.17 ^c^

MW: minimum wages; SD: standard deviation; Min: minimum value; Max: maximum value; sk: skewmess; ku: kurtosis; ECC: Brazilian Economic Classification Criteria; SBP: systolic blood pressure; DBP: diastolic blood pressure; WHR: waist-to-height ratio; BMI: body mass index; WC: waist circumference.

^a^ Values corrected by drawing effect (deff).

^b^ Mean of the three measurements.

^c^ Values obtained after logarithmic transformation.

**Figure 2:**
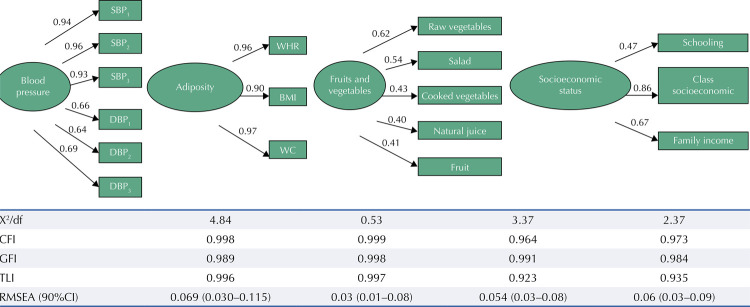
Adjusted measurement models, with their respective adjustment indices, for the constructs blood pressure, adiposity, fruit and vegetable consumption and socioeconomic status in adults (≥ 18 years). Montes Claros, Minas Gerais, 2012-2013 (n = 808).

SBP: systolic blood pressure; DBP: diastolic blood pressure; WHR: waist-to-height ratio; BMI: body mass index; WC: waist circumference; X ^2^: chi-square; df: degrees of freedom; CFI: Bentler’s comparative adjustment index; GFI: goodness of fit index; TLI: Tucker-Lewis index; RMSEA: root mean square error approximation.

Figure 2 shows the results of the confirmatory factor analysis that operationalized the latent variables BP, adiposity, fruit and vegetable consumption and socioeconomic status. Most of the observed variables that composed each of these constructs presented adequate factor weights (≥ 0.5) and all were significant. The goodness of fit indices for the model adjustments presented values considered acceptable.

**Figure 3:**
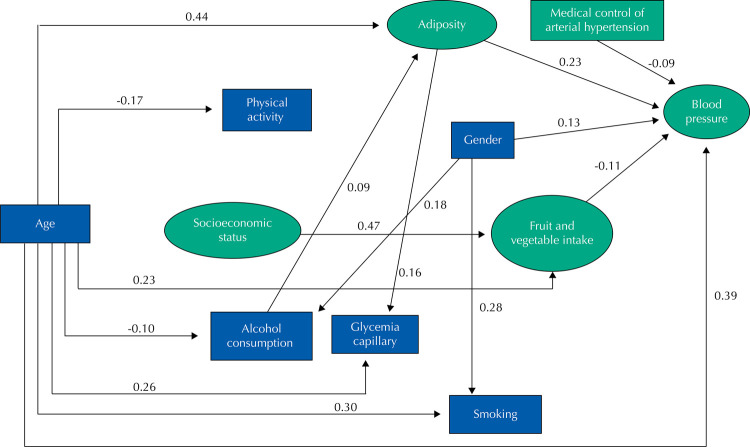
Structural equation model adjusted to evaluate the interrelations between blood pressure, age, socioeconomic status, physical activity, alcohol consumption, smoking, fruit and vegetable intake, adiposity, capillary glycemia and medical control of arterial hypertension in adults (≥ 18 years). Montes Claros, Minas Gerais, 2012–2013 (n = 808).

Note: All values were significant at the level of 0.05.

Figure 3 shows the adjusted structural model, whose fit indices were considered acceptable when X ^2^ /df = 3.79; CFI = 0.960; GFI = 0.953; TLI = 0.947; RMSEA = 0.052 (90% CI 0.047–0.057). It shows only the standardized structural coefficients that showed statistical significance of 0.05 level. According to the model, age exerted a direct positive effect on BP (β = 0,39; p < 0.001), adiposity (β = 0,44; p < 0.001), glycemia (β = 0,26; p < 0,001), fruit and vegetable intake (β = 0,23; p < 0.001), and smoking (β = 0,30; p < 0.001); and direct negative effect on physical activity (β = -0,17; p < 0.001), and alcohol consumption (β = -0,10; p = 0.042). Males were positively associated with BP (β = 0.13; p = 0.040), smoking (β = 0.28; p < 0.001) and alcohol consumption (β = 0.18; p < 0.001). Adiposity had a direct positive effect on BP (β = 0.23; p < 0.001) and glycemia (β = 0.16; p < 0.001), and that alcohol consumption had a positive effect (β = 0.09; p = 0.005) on adiposity. We observed a direct negative effect of fruit and vegetable consumption on BP (β = -0.11; p < 0.001) and a direct positive effect of socioeconomic status on fruit and vegetable consumption (β = 0.47; p < 0.001). Medical control of arterial hypertension had a direct negative effect on blood pressure levels (β = -0.09; p = 0.044).

In the mediation analysis, most indirect effects showed no statistical significance, except the indirect effect of age on BP (β = 0.1012; p = 0.040), mediated mainly by adiposity. Table 2 shows the magnitudes of direct, indirect and total effects.

**Table 2:** Magnitude of direct, indirect and total effects among factors influencing blood pressure in adults (≥ 18 years). Montes Claros, Minas Gerais, 2012–2013.

Independent variable	Effect	Dependent variable	Coefficient	
	Direct/indirect		Direct/indirect	Total
Age	Direct 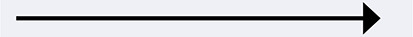	Blood pressure	0.39	0.49
	Via adiposity (indirect) 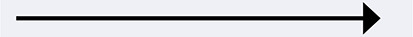		0.44 × 0.23 = 0.1012	
Gender	Direct 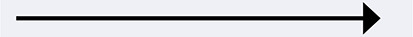	Blood pressure	0.13	0.13
	Direct	Alcohol consumption	0.18	0.18
	Direct 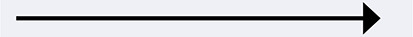	Smoking	0.28	0.28
Age	Direct	Adiposity	0.44	0.44
	Direct 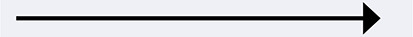	Glycemia	0.27	0.27
	Direct 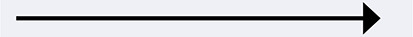	Smoking	0.30	0.30
	Direct 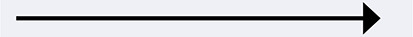	Fruit and vegetable intake	0.23	0.23
	Direct 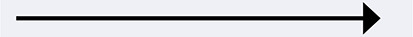	Alcohol consumption	-0.10	-0.10
	Direct 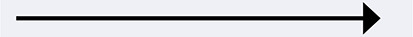	PA practice	-0.17	-0.17
Adiposity	Direct 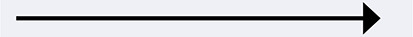	Blood pressure	0.23	0.23
	Direct 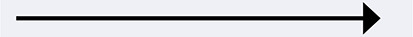	Glycemia	0.16	0.16
SES	Direct 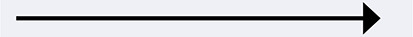	Fruit and vegetable intake	0.47	0.4
Alcohol consumption	Direct 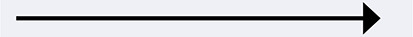	Adiposity	0.09	0.09
Fruit and vegetable intake	Direct 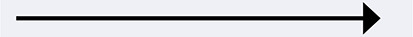	Blood pressure	-0.11	-0.11
Medical control of SAH	Direct 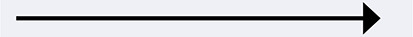	Blood pressure	-0.09	-0.09

SES: socioeconomic status; SAH: systemic arterial hypertension; PA: physical activity

## DISCUSSION

This study investigated the interrelations between BP, age, gender, socioeconomic status, PA, alcohol consumption, smoking, fruit and vegetable intake, adiposity and capillary glycemia via SEM. By adopting this multivariate technique, we were able to analyze a system composed of multiple factors and visualize their interrelations.

On the adjusted model, we identified that age plays an important role in this system of interrelations. Age had a significant effect on BP, adiposity, glycemia, fruit and vegetable consumption, smoking, PA and alcohol consumption, being the factor with the greatest effect on BP. Such association is well described in the literature, which report an increase in the prevalence of SAH with advancing age ^3 , 6 , 14^ , factor that brings intrinsic changes to the aging process, such as hardening of the arteries and increased peripheral vascular resistance, which affects PA ^14^ . Our results also showed this indirect effect of age on blood pressure levels, mediated mainly by adiposity.

In addition, the study found that advancing age correlated with the increase in adiposity, corroborating results from another study, which observed an increase in the prevalence of body and central obesity with time ^15^ . In this context, changes such as reduced lean mass, increased body fat percentage, decreased height, relaxation of abdominal muscles, changes in BMI and RCE ^16^ are observed. However, it is noteworthy that, unlike what occurs in adults, in older adults, weight decreases with age after reaching a plateau around 65 and 75 years in men and women, respectively.

This study also found that advancing age was associated with increased glycemic levels. This finding is reflected in the increased prevalence of diabetes with the advancing age of a population ^17^ . In this sense, the demographic transition observed in Brazil in recent decades has been identified as one of the main causes of the increase in the incidence and prevalence of diabetes in the country ^18^ . Although unhealthy habits and behaviors such as sedentary lifestyle and inadequate nutrition also contribute to metabolic alterations ^19^ , this study found no effects of these factors on glycemic levels.

We observed an effect of age on health-related behaviors such as smoking, fruit and vegetable consumption, PA and alcohol consumption. There was a positive effect of age on smoking, as observed in previous studies ^20^ , indicating that older individuals consume more cigarettes than younger ones. The campaigns to control smoking in Brazil articulated by the Ministry of Health, intensified since the late 1980s, may explain this finding. With a greater success among younger individuals, these campaigns helped hinder the smoking habit between this population, while older and smokers persist with this practice.

Smoking has been considered a strong predictor for developing arterial hypertension. Paradoxically, the present study found no positive relationship between blood pressure levels and smoking. Thus, the adjusted model established no correlation between the increase in the number of cigarettes consumed and the increase in blood pressure levels, regardless of age. Such fact can be explained by the numerical and continuous nature in which we measured the variable smoking (pack-year), which probably hindered identifying its effect on blood pressure levels, since nearly 77% of interviewees reported having never smoked (pack-year = 0). Another aspect to be considered is the tendency of blood pressure levels normalizing a one hour after cigarette consumption, which may have occurred with the smokers in the sample.

Regarding PA, age had a small negative effect, suggesting that advancing age is associated with a reduction in weekly practice time. This correlation may partially result from inequality in access, supply and use of resources, equipment and services for practicing PA, since this behavior can be influenced by environmental and individual characteristics. A study ^21^ conducted in the city of São Paulo found a decrease in the frequency of PA according to age progression. However, the frequency was higher among individuals aged 50 to 59 years, than among those aged 30 to 39 and 40 to 49 years. Individuals aged 50 to 59 years may have more opportunities to practice PA or need to perform it for health promotion, for minimizing the progression of common chronic diseases in the aging process ^22^ .

The same trend was observed in the effect of age on alcohol consumption, i.e., advancing age was correlated with lower alcohol consumption. Although of a small effect, this result corroborates the findings of a previous study ^23^ . In this sense, we have a trend of higher alcohol consumption among young people, which, according to the WHO ^24^ , reflects a worldwide pattern. In adolescence and early adulthood, group meetings intensify, and alcohol consumption tends to increase, which can lead to abuse. Thus, adolescents and young adults constitute the population at greatest risk of alcohol consumption ^25^ . On the other hand, a previous study ^21^ observed an increase in alcohol consumption with age progression, a result of the increased life expectancy and population’s income. It is worth noting, however, that 66% of the respondents in this study reported not consuming alcohol.

We also observed a positive effect of adiposity on BP in the adjusted model, corroborating previous studies ^3 , 5^ that observed an association between increased BMI and prevalence of SAH. According to the Brazilian Society of Hypertension (SBH) ^4^ , 75% of men and 65% of women have SAH directly attributed to excess weight. The mechanisms by which the increased body mass interferes with the change in BP levels still require clarification. However, this association can be attributed to the accumulation of intra-abdominal fat that contributes to greater sympathetic activity, which in turn would increase sodium reabsorption, resulting in an increase in peripheral vascular resistance and, consequently, BP. Moreover, intra-abdominal fat also increases pro-inflammatory cytokines, which may interfere in high blood pressure values ^26^ .

This study found a positive effect of adiposity on capillary glycemia, although the effect was of low magnitude, as observed in other studies ^17^ . This association can be explained by the process of accumulation of fat in the liver, which affects liver metabolism, increasing insulin resistance. In addition, excess circulating fat and glucose increases the demand for insulin secretion by the pancreas, leading the insulin-producing cells to stress and exhaustion ^19^ .

Our findings also identified the positive effect of alcohol consumption on adiposity. Although small, the effect was consistent with that observed in another study ^27^ . Alcoholic beverages are highly caloric and, added to the normal diet, can contribute to metabolic and hormonal changes, with repercussions on energy homeostasis, affecting appetite and weight gain ^27^ .

There was a small negative effect of fruit and vegetable consumption on BP. Results show that a higher consumption of fruits and vegetables was associated with lower blood pressure levels, as observed in a previous study ^5^ . Adequate consumption of fruits and vegetables favors BP reduction due to the high concentration of minerals with hypotensive potential in these foods, especially magnesium and potassium, and for their low fat content ^5^ .

The adjusted model identified that males had higher blood pressure levels, which may be related to this population’s greater exposure to a greater number of risk factors, such as low schooling level, alcohol consumption and low demand for health services ^4^ . The study results also showed that men made greater use of alcohol and cigarettes compared with the women.

The adjusted model pointed out that the better socioeconomic status was associated with a higher frequency of fruit and vegetable consumption, association consistent with another study ^28^ . Indeed, social determinants such as schooling are related with accumulation of knowledge, able to increase health care and healthy habits. As such, people with a higher schooling level show a more critical perception about their health and tend to adopt a more careful behavior with it. Higher schooling level also enables better working conditions and income, which affects access to healthier foods.

Eating foods rich in fat and sodium may influence high blood pressure levels ^1 , 3^ ; however, this study did not assess these parameters, thus constituting a limitation. Another limitation arises from the lack of information about family history, since estimates of heritability have shown that 15% to 60% of BP variation can be attributed to genetic factors ^29^ .

The direct analysis of data from a cluster sample, with the census sector being the primary sampling unit and not the individual, presented another limitation. This type of sample requires a *survey* statistical analysis, which we did not perform in the model adjustment. When performing the direct analysis, there is a risk of obtaining spurious associations due to the overestimation of the fit quality provided by the artificial increase in the number of degrees of freedom. Therefore, we recommend caution in interpreting the associations identified. Another limitation of this study is its cross-sectional nature, with pressure levels and other variables used in the model measured simultaneously, being impossible to determine the temporal sequence of events. Thus, although the adjusted model allowed to raise important interrelation hypotheses, it did not confirm causality.

Once the relationship between BP, adiposity, glycemia, age, gender and health-related behaviors has been established, it can be assumed that high blood pressure may become progressively more prevalent in the absence of changes in population behavior, since age is a non-modifiable factor. Therefore, given the Brazilian population’s aging trend, actions at the individual and population levels aimed at health promotion are necessary, such as interventions that stimulate healthy eating, regular PA and restrictions on alcohol consumption and smoking, which probably would have an important impact on reducing the prevalence of high BP.
