# Supplementary material for: Analysis of the interrelationships between factors influencing blood pressure in adults
Source: Rev Saude Publica. 2020 Dec 4;54:147. doi: 10.11606/s1518-8787.2020054002123 (PMC7726937; doi:10.11606/s1518-8787.2020054002123)
Supplement: Translation

# Translation: Análise das inter-relações entre os fatores que influenciam a pressão arterial em adultos

**Authors:** Rafael Silveira Freire, Vivianne Margareth Chaves Pereira Reis, Alexandre Botelho Brito, Maria Fernanda Santos Figueiredo Brito, Lucinéia de Pinho, Rosângela Ramos Veloso Silva, Marise Fagundes Silveira

**Affiliations:** I Brasil Faculdades Unidas do Norte de Minas. Instituto de Ciências da Saúde. Montes Claros, MG, Brasil; II Brasil Universidade Estadual de Montes Claros. Departamento de Educação Física. Montes Claros, MG, Brasil; III Brasil Instituto Federal do Norte de Minas Gerais. Departamento de Matemática. Montes Claros, MG, Brasil; IV Brasil Universidade Estadual de Montes Claros. Programa de Pós-Graduação em Cuidado Primário em Saúde. Montes Claros, MG, Brasil; V Brasil Universidade Estadual de Montes Claros. Programa de Pós-Graduação em Ciências da Saúde. Montes Claros, MG, Brasil

**Keywords:** Adulto, Pessoa de Meia-Idade, Hipertensão, epidemiologia, Comportamentos Relacionados com a Saúde, Fatores de Risco, Fatores Socioeconômicos, Estudos Transversais

## Abstract

**OBJETIVO:**

Investigar as inter-relações entre fatores sociodemográficos, comportamentais e clínicos associados à elevação da pressão arterial em adultos.

**MÉTODOS:**

Utilizaram-se dados de um estudo transversal de base populacional. Foi desenvolvido um modelo hipotético em que condição socioeconômica, consumo de frutas e vegetais, adiposidade e pressão arterial foram tratadas como variáveis latentes, e idade, sexo, glicemia, atividade física, tabagismo, consumo de álcool e controle da hipertensão arterial (HA) foram tratadas como variáveis observadas. Utilizou-se a modelagem de equação estrutural para ajustar o modelo final.

**RESULTADOS:**

Participaram do estudo 808 indivíduos, com idade média de 44,2 anos (DP = 17,8), sendo 52,7% do sexo feminino. Verificou-se que a idade exerceu efeito positivo sobre pressão arterial (β = 0,39), adiposidade (β = 0,44), glicemia (β = 0,26) e tabagismo (β = 0,30), e efeito negativo sobre atividade física (β = -0,17) e consumo de álcool (β = -0,09). O sexo masculino mostrou-se associado positivamente com a pressão arterial (β = 0,13), com o tabagismo (β = 0,28) e com o consumo de bebida alcoólica (β = 0,18). A adiposidade teve efeito positivo sobre a pressão arterial (β = 0,23) e a glicemia (β = 0,16), o consumo de álcool produziu efeito positivo sobre a adiposidade (β = 0,09), o consumo de frutas e vegetais exerceu efeito negativo sobre a pressão arterial (β = -0,11), e a condição socioeconômica teve efeito positivo sobre o consumo de frutas e vegetais (β = 0,47). O modelo estrutural foi ajustado pela variável controle médico da hipertensão, que apresentou efeito negativo sobre a pressão arterial (β = -0,10).

**CONCLUSÕES:**

Os resultados sugerem que o aumento da idade se associa ao aumento da pressão arterial, adiposidade, glicemia e tabagismo, assim como com a redução da atividade física e consumo de álcool. O sexo masculino associou-se com aumento da pressão arterial e maior uso de bebida alcoólica e cigarros. A adiposidade correlaciona-se ao aumento da pressão arterial e da glicemia, enquanto maior consumo de álcool associa-se com aumento da adiposidade e maior consumo de frutas e vegetais, assim como o controle ativo da hipertensão, correlaciona-se com redução da pressão arterial. A melhor condição socioeconômica mostrou-se associada com maior consumo de frutas e vegetais.

## INTRODUÇÃO

As doenças cardiovasculares (DCV) são as principais causas de morbidade e mortalidade em todo o mundo, sendo responsáveis por cerca de 17 milhões de mortes por ano. Dessas, aproximadamente 55% são complicações decorrentes da hipertensão arterial sistêmica (HAS), constituindo-se em uma das dez maiores causas de óbito no mundo^1^ .

A prevalência global de HAS em adultos foi estimada em 22%, registrando-se prevalências de maior magnitude em regiões da África (30%), enquanto nas Américas, Europa, Ásia e Oceania, as prevalências oscilaram entre 18% e 25%^2^ . No Brasil, a HAS é considerada um dos mais relevantes problemas de saúde pública, com uma prevalência de aproximadamente 30% na população adulta, chegando a mais de 50% entre 60 e 69 anos e de 75% acima de 70 anos^3^ .

Diante das expressivas prevalências e taxa de mortalidade atribuível à HAS na população global, observa-se na literatura um número significativo de pesquisas que buscam estimar os efeitos dos fatores a ela associados, incluindo os genéticos, ambientais e comportamentais^4^ . No entanto, essa questão tem sido mais comumente analisada utilizando-se técnicas clássicas de estatística, que se caracterizam por contemplar múltiplos fatores de risco, mas limitam-se a uma única relação entre eles e o desfecho.

Por outro lado, a elevação da pressão arterial (PA) é um fenômeno que sofre influência de um conjunto de fatores que se inter-relacionam^3^ . Alguns desses fatores, como obesidade e diabetes mellitus, atuam diretamente, enquanto outros agem indiretamente por meio de fatores intermediários, incluindo hábitos e comportamentos como consumo excessivo de álcool, tabagismo, inatividade física ou alimentação inadequada. Os fatores de risco mais distais, como escolaridade e renda, possuem associações diretas menos evidentes. No entanto, modificações de fatores intermediários ou distais podem influenciar os fatores de risco proximais e resultarem em maior magnitude de efeito sobre a elevação da PA. Portanto, verifica-se que existe uma complexa rede de fatores inter-relacionados que influenciam os níveis pressóricos e que podem ser analisados simultaneamente para melhor compreensão desse fenômeno e das relações existentes entre as variáveis. Entretanto, não foram identificados outros estudos que investigaram essas inter-relações utilizando-se a modelagem de equações estruturais.

Nesse contexto, o presente estudo teve como objetivo investigar as inter-relações entre os fatores sociodemográficos, comportamentais e clínicos associados à elevação da PA em uma população de adultos brasileiros.

## MÉTODOS

Foram utilizados dados do estudo intitulado “Polimorfismo do gene do receptor da leptina (rs1137101), obesidade e sua associação com fatores de risco para as doenças cardiovasculares em Montes Claros, Minas Gerais”^7^ . Trata-se de um estudo epidemiológico, transversal, de base populacional realizado em indivíduos com idade igual ou superior a 18 anos, residentes na região urbana do município de Montes Claros, Minas Gerais, Brasil, no ano de 2013.

O cálculo do tamanho amostral, conforme descrito por Pena et al.^7^ , estimou a participação de no mínimo 750 adultos. Para tal, considerou-se frequência esperada de 10% do polimorfismo do receptor de leptina, coeficiente de variação inferior a 25%, erro-padrão menor que 3% e correção para o efeito do desenho ( *deff* ) igual a 2,0.^6^ O supracitado tamanho amostral definido para a pesquisa atende às premissas para o tamanho amostral da presente investigação, a qual, visando estimar prevalência de hipertensão arterial igual a 30%^2 , 4^ , ao nível de confiança de 95%, margem de erro absoluto de 5%, com correção para o efeito do desenho 2,0 e um acréscimo de 10% para a taxa de não-resposta, estimou um tamanho mínimo de 710 indivíduos.

Foi adotada amostragem probabilística por conglomerado em dois estágios. No primeiro estágio foram sorteados os setores censitários, e no segundo, os domicílios. Previamente à coleta de dados, foram realizadas capacitação e calibração dos pesquisadores, além de ter sido conduzido um estudo-piloto com uma amostra de conveniência.

A coleta dos dados foi realizada nos domicílios selecionados, nos quais foi utilizado um questionário estruturado constituído por variáveis relacionadas às características sociodemográficas e comportamentos relacionados à saúde. Foi também realizada a aferição de variáveis antropométricas, PA e glicemia capilar^7^ .

As características sociodemográficas incluíram sexo, idade, escolaridade, classe socioeconômica e renda familiar. A escolaridade foi avaliada considerando a última série de estudo cursada pelo indivíduo e classificada em 10 categorias ordinais (1: analfabeto; 2: 4ª série incompleta; 3: 4ª série completa; 4: 8ª série incompleta; 5: 8ª série completa; 6: ensino médio incompleto; 7: ensino médio completo; 8: ensiono superior incompleto; 9: ensino superior completo e 10: pós-graduação). Para avaliar a classe socioeconômica, foi utilizado o sistema de pontuação do Critério de Classificação Econômica Brasil, cuja escala varia de 0 a 46 pontos, sendo que quanto maior a pontuação, melhor a classificação socioeconômica dos indivíduos^8^ . A renda familiar foi expressa por salários mínimos (SM), sendo o salário vigente de R$ 622,00. Essa variável foi dividida em seis categorias ordinais (1: < 1 SM; 2: 1 a 1,99 SM; 3: 2 a 3,99 SM; 4: 4 a 5,99 SM; 5: 6 a 7,99 SM; e 6: ≥ 8 SM).

Os comportamentos relacionados à saúde foram avaliados por meio das variáveis prática de atividade física (AF), consumo de frutas e vegetais, consumo de bebida alcoólica e tabagismo. Para mensurar a prática de AF, foi adotado o Questionário Internacional de Atividade Física (IPAQ) na versão longa^9^ . Calculou-se o tempo semanal (em minutos) de prática de caminhada e de atividade física moderada e vigorosa nos domínios do trabalho, meio de transporte, do lar (atividades domésticas) e recreação, esporte, exercício e lazer. O consumo de frutas e vegetais foi avaliado pela frequência semanal de consumo de verdura crua, salada, verdura cozida, suco natural e fruta. A categorização dessas variáveis foi expressa pela seguinte escala ordinal: 0: nunca/quase nunca; 1: um a dois dias por semana; 2: três a quatro dias por semana; 3: cinco a seis dias por semana e 4: todos os dias na semana. O consumo de bebida alcoólica foi calculado multiplicando-se a frequência semanal pelo número de doses ingeridas em cada ocasião. O tabagismo foi medido em maços-ano, que representa o produto do número de maços fumados por dia (1 maço = 20 cigarros) pelo número de anos em que o indivíduo fumou.

As medidas antropométricas foram realizadas em duplicada e incluíram peso (em quilogramas), altura (em metros) e circunferência da cintura (CC) (em centímetros). As técnicas empregadas para obtenção dessas medidas seguiram os protocolos da Organização Mundial de Saúde (OMS)^10^ . O peso corporal foi medido utilizando uma balança portátil (modelo PL 150, GTech) com uma precisão de 0,1 kg. A altura foi mensurada por um estadiômetro portátil (Alturexata) com base de metal e aparato para nivelamento da altura funcionando como o esquadro com precisão de 0,1 cm. Para aferição da CC, utilizou-se uma fita métrica inelástica (TBW), com precisão de 1 mm. Foi calculado o índice de massa corporal (IMC) de acordo com a fórmula: IMC = peso (kg)/estatura^2^(m). Calculou-se também a razão cintura-estatura (RCE), dividindo-se a medida da CC pela medida da altura, ambas em centímetros.

A PA foi aferida utilizando o esfigmomanômetro digital calibrado (modelo HEM-7200, Omron), conforme o protocolo das Diretrizes Brasileiras de Hipertensão Arterial^11^ , sendo realizadas três aferições com o intervalo de pelo menos cinco minutos. Foram também coletadas informações sobre a realização de controle médico da hipertensão arterial, por meio das seguintes perguntas: “Alguma vez o médico já disse que o(a) Sr.(a) tem hipertensão ou pressão alta? (sim ou não)” e “Se sim, você faz controle médico? (sim ou não)”. A aferição da glicemia capilar foi realizada pela técnica de punção capilar, na qual se utilizou o glicosímetro digital (modelo Accu-Check^®^Performa, Roche Diagnostics).

Condição socioeconômica, consumo de frutas e vegetais, adiposidade e PA foram tratadas como variáveis latentes. A condição socioeconômica foi definida por três variáveis observadas: escolaridade, classe socioeconômica e renda familiar. O consumo de frutas e vegetais foi constituído pelas variáveis: consumo de verdura crua, salada, verdura cozida, suco natural e fruta. A adiposidade foi medida pelas variáveis IMC, CC e RCE na primeira medição e a PA pelos valores da pressão arterial sistólica (PAS) e pressão arterial diastólica (PAD) nas três medições.

Foi elaborado um modelo teórico multivariado, adaptado do modelo conceitual de fatores de risco para doença arterial coronariana. Buscou-se avaliar as inter-relações entre a PA, considerada como desfecho principal, e as demais variáveis do estudo: sexo, idade, condição socioeconômica, consumo de frutas e vegetais, prática de AF, consumo de bebida alcoólica, tabagismo, adiposidade, glicemia capilar e controle médico da hipertensão arterial. A Figura 1 ilustra as relações diretas e indiretas entre as variáveis investigadas no modelo proposto. As variáveis observadas estão representadas por retângulos, os construtos por elipses, e as correlações representadas por setas (da variável independente para a dependente)^12^ .

**Figura 1:** Modelo hipotético testado para avaliar as inter-relações entre os fatores que influenciam a pressão arterial em adultos (≥ 18 anos). Montes Claros, Minas Gerais, 2012–2013 (n = 808).

RCE: razão cintura-estatura; IMC: índice de massa corporal; CC: circunferência da cintura; PAS: pressão arterial sistólica; PAD: pressão arterial diastólica

Na análise dos dados, as variáveis categóricas foram descritas por meio de suas distribuições de frequências, e as variáveis numéricas por média, desvio-padrão (DP), valores mínimo e máximo, coeficientes de assimetria (sk) e curtose (ku), com correção pelo efeito do desenho ( *deff* ), por serem provenientes de uma amostra por conglomerado. Para incorporar a estrutura do plano amostral complexo na análise descritiva das variáveis e corrigir a perda de precisão das estimativas, cada entrevistado foi associado a um peso, que correspondeu ao inverso de sua probabilidade de inclusão na amostra. Valores de sk > 3 e ku > 10 foram considerados como indicadores de violação do pressuposto da normalidade^12^ . As variáveis consumo de bebida alcoólica, prática de AF, tabagismo e glicemia capilar receberam transformação logarítmica por violarem a suposição de normalidade. Os valores perdidos foram imputados pela média.

O ajuste do modelo foi realizado em duas etapas. Inicialmente foram ajustados os modelos de mensuração, que estabeleceram como as variáveis latentes foram mensuradas. Para tal, utilizou-se a análise fatorial confirmatória (AFC). Considerou-se como variável latente bem ajustada aquela que apresentou todos os pesos fatoriais significantes (p < 0,05) e pesos fatoriais padronizados maiores ou iguais a 0,50 (λ ≥ 0,50)^12^ .

Posteriormente, foi ajustado o modelo multivariado por meio da modelagem de equações estruturais (MEE). Foram estimados efeitos diretos e indiretos representados por coeficientes padronizados, cujas significâncias estatísticas foram avaliadas pela razão crítica (RC), ao nível de 5%. Os coeficientes estruturais padronizados expressam a variação, em desvio-padrão, na variável dependente por variação de uma unidade do desvio-padrão na variável independente. Quando positivos, indicam que as variáveis “caminham” no mesmo sentido, ou seja, o aumento na variável independente está correlacionado com o aumento na variável dependente. Por outro lado, quando o coeficiente é negativo, indica que o aumento na variável independente está correlacionado com redução nos valores da variável independente. Coeficientes padronizados com valores próximos de 0,10, próximos de 0,30 e superiores a 0,50 foram interpretados como efeitos pequeno, médio e grande, respectivamente^12^ .

Na avaliação da qualidade de ajuste dos modelos, foram utilizados os índices de ajuste comparativo de Bentler (CFI), de adequação de ajuste (GFI) e de Tucker-Lewis (TLI), tendo-se considerado que indicam um bom ajuste para valores superiores a 0,90^13^ . Utilizou-se também a raiz do erro quadrático médio de aproximação (RMSEA), cujos valores inferiores a 0,10 foram considerados indicadores de ajuste razoável. O índice absoluto X^2^/gl também foi adotado, tendo-se considerado que indica um ajuste aceitável para valor inferior a 5^13^ .

Para as análises descritivas, foi utilizado o módulo *complex sample* implementado no software IBM SPSS 23.0. Os modelos de mensuração e estrutural foram ajustados utilizando-se o software IBM SPSS Amos 23.0 e o software R 3.5.0, respectivamente.

Todos os participantes assinaram o Termo de Consentimento Livre e esclarecido. O projeto de pesquisa que originou o presente estudo foi aprovado pelos comitês de ética em pesquisa da Universidade Estadual de Montes Claros (parecer nº 226.604/2013) e da Universidade Federal de Minas Gerais (parecer nº 213.555/2013).

## RESULTADOS

Participaram do estudo 808 indivíduos, cuja média de idade foi 44,2 anos (DP = 17,8), sendo 52,7% do sexo feminino. A renda familiar mensal foi inferior a dois salários mínimos para aproximadamente metade (46%) dos entrevistados, e 39,5% apresentaram escolaridade inferior ao ensino médio. A PAS média foi de 129,5 mmHg (DP = 21,3) e a PAD média foi de 76,1mmHg (DP = 12,0). Dos investigados, 26,3% apresentaram PA elevada (PAS ≥ 140 mmHg e/ou PAD ≥ 90 mmHg) e 17,7% relataram fazer controle médico da hipertensão arterial. Na Tabela 1 estão apresentadas as medidas descritivas das demais características dos participantes.

**Tabela 1:** Variáveis relacionadas às condições sociodemográficas, aspectos comportamentais e clínicos em adultos (≥ 18 anos). Montes Claros, Minas Gerais, 2012–2013.

Variável	n	%^a^	Variável		n	%^a^
**Escolaridade (n = 806)**			**Consumo de salada (n = 808)**			
Analfabeto	62	5,5	1 a 2 dias/semana		28	31,7
4ª série imcompleta	99	93,7	3 a 4 dias/semana		85	11,7
4ª série completa	101	103,8	5 a 6 dias/semana		194	25,5
8ª série incompleta	54	7,1	5 a 6 dias/semana		158	18,9
8ª série completa	56	6,4	Todos os dias		343	40,2
Ensino médio incompleto	60	9,3	**Consumo de verdura cozida (n = 797)**			
Ensino médio completo	260	36.3	Nunca/quase nunca		89	11,0
Ensino superior incompleto	58	8,4	1 a 2 dias/semana		249	31,6
Ensino superior completo	48	5,6	3 a 4 dias/semana		220	29,3
Pós-graduação	9	0,9	5 a 6 dias/semana		130	15,9
**Renda em SM (n = 807)**			Todos os dias		109	12,1
Menos de 1	23	3,4	**Consumo de suco natural (n = 808)**			
De 1 a 1,99	347	42,6	Nunca/quase nunca		211	28,2
De 2 a 3,99	348	43,2	1 a 2 dias/semana		237	28,4
De 4 a 5,99	46	5,9	3 a 4 dias/semana		188	24,2
De 6 a 7,99	23	3,0	5 a 6 dias/semana		95	10,5
8 ou mais	20	1,9	Todos os dias		77	8,8
**Consumo de verdura crua (n = 794)**			**Consumo de frutas (n = 808)**			
Nunca/quase nunca	92	10,9	Nunca/quase nunca		69	8,7
1 a 2 dias/semana	243	31,0	1 a 2 dias/semana		189	26,6
3 a 4 dias/semana	193	25,4	3 a 4 dias/semana		203	24,2
5 a 6 dias/semana	124	15,2	5 a 6 dias/semana		133	15,4
Todos os dias	142	17,4	Todos os dias		214	25,1

**Variáveis**	**Média (DP)^a^**		**Mín**	**Máx**	**sk**	**ku**

Idade	44,2 (17,8)		18,0	91,0	0,44	-0,61
Escore CCE	18,0 (5,8)		6,0	42,0	1,03	1,40
PAS^b^	129,5 (21,3)		86,7	209,3	0,88	0,77
PAD^b^	76,1 (12,0)		46,7	122,0	0,50	0,30
RCE	0,54 (0,09)		0,36	0,87	0,40	0,09
IMC	26,2 (5,6)		14,9	53,8	0,96	1,73
CC	87,3 (13,7)		58,0	143,0	0,47	0,33
Glicemia capilar	121,7 (53,9)		58,0	600,0	2,28^c^	7,68^c^
Atividade física	800,5 (834,0)		0,0	1.960	1,52^c^	2,08^c^
Consumo de bebida alcoólica	4,3 (9,8)		0,0	41,0	1,90^c^	2,48^c^
Tabagismo	4,8 (13,8)		0,0	120,0	1,89^c^	2,17^c^

SM: salários mínimos; DP: desvio-padrão; Mín: valor mínimo; Máx: valor máximo; sk: assimetria; ku: curtose; CCE: Critério de Classificação Econômica Brasil; PAS: pressão arterial sistólica; PAD: pressão arterial diastólica; RCE: razão cintura-estatura; IMC: índice de massa corporal; CC: circunferência da cintura.

^a^Valores corrigidos pelo efeito do desenho (deff).

^b^Média das três medições.

^c^Valores obtidos após a transformação logarítmica.

A Figura 2 mostra os resultados da análise fatorial confirmatória que operacionalizou as variáveis latentes PA, adiposidade, consumo de frutas e vegetais e condição socioeconômica. A maioria das variáveis observadas que compôs cada um destes construtos apresentou pesos fatoriais adequados (≥ 0,5) e todos foram significativos. Os índices de qualidade de ajuste dos modelos apresentaram valores considerados aceitáveis.

**Figura 2:** Modelos de mensuração ajustados, com seus respectivos índices de ajuste, para os construtos pressão arterial, adiposidade, consumo de frutas e vegetais e condição socioeconômica em adultos (≥ 18 anos). Montes Claros, Minas Gerais, 2012–2013 (n = 808).

PAS: pressão arterial sistólica; PAD: pressão arterial diastólica; RCE: relação cintura-estatura; IMC: índice de massa corporal; CC: circunferência de cintura; X^2^: qui-quadrado; gl: graus de liberdade; CFI: índice de ajuste comparativo de Bentler; GFI: índice de adequação de ajuste; TLI: índice de Tucker-Lewis; RMSEA: raiz do erro quadrático médio de aproximação.

O modelo estrutural ajustado, cujos índices de ajuste foram considerados aceitáveis quando X^2^ /gl = 3,79; CFI = 0,960; GFI = 0,953; TLI = 0,947; RMSEA = 0,052 (IC90% 0,047–0,057), está apresentado na Figura 3 . Nela estão exibidos apenas os coeficientes estruturais padronizados que apresentaram significância estatística ao nível de 0,05. De acordo com o modelo, a idade exerceu efeito direto positivo sobre PA (β = 0,39; p < 0,001), adiposidade (β = 0,44; p < 0,001), glicemia (β = 0,26; p < 0,001), consumo de frutas e vegetais (β = 0,23; p < 0,001) e tabagismo (β = 0,30; p < 0,001), e efeito direto negativo sobre atividade física (β = -0,17; p < 0,001) e consumo de bebida alcóolica (β = -0,10; p = 0,042). O sexo masculino mostrou-se associado positivamente com a PA (β = 0,13; p = 0,040), com o tabagismo (β = 0,28; p < 0,001) e com o consumo de bebida alcoólica (β = 0,18; p < 0,001). Constatou-se que a adiposidade exerceu efeito direto positivo sobre a PA (β = 0,23; p < 0,001) e a glicemia (β=0,16; p < 0,001), e que o consumo de bebida alcoólica teve efeito positivo (β = 0,09; p = 0,005) sobre a adiposidade. Observou-se efeito direto negativo do consumo de frutas e vegetais sobre a PA (β = -0,11; p < 0,001) e efeito direto positivo da condição socioeconômica sobre o consumo de frutas e vegetais (β = 0,47; p < 0,001). O controle médico da hipertensão arterial exerceu efeito direto negativo sobre os níveis pressóricos (β = -0,09; p = 0,044).

**Figura 3:** Modelo de equações estrutural ajustado para avaliar as inter-relações entre pressão arterial, idade, condição socioeconômica, prática de atividade física, consumo de bebida alcoólica, tabagismo, consumo de frutas e vegetais, adiposidade, glicemia capilar e controle médico da hipertensão arterial em adultos (≥ 18 anos). Montes Claros, Minas Gerais, 2012–2013 (n = 808).

Nota: todos os valores foram significantes ao nível de 0,05.

Na análise de mediação, a maioria dos efeitos indiretos não se mostrou estatisticamente significativa, com exceção do efeito indireto da idade sobre a PA (β = 0,1012; p = 0,040), mediado principalmente pela adiposidade. As magnitudes dos efeitos direto, indireto e total estão apresentadas na Tabela 2 .

**Tabela 2:** Magnitude dos efeitos direto, indireto e total entre os fatores que influenciam a pressão arterial em adultos (≥ 18 anos). Montes Claros, Minas Gerais, 2012–2013.

Variável independente	Efeito	Variável dependente	Coeficiente	
	Direto/indireto		Direto/indireto	Total
Idade	Direto	Pressão arterial	0,39	0,49
	Via adiposidade (indireto)		0,44 × 0,23 = 0,1012	
Sexo	Direto	Pressão arterial	0,13	0,13
	Direto	Consumo de bebida alcóolica	0,18	0,18
	Direto	Tabagismo	0,28	0,28
Idade	Direto	Adiposidade	0,44	0,44
	Direto	Glicemia	0,27	0,27
	Direto	Tabagismo	0,30	0,30
	Direto	Consumo de frutas e vegetais	0,23	0,23
	Direto	Consumo de bebida alcóolica	-0,10	-0,10
	Direto	Prática de AF	-0,17	-0,17
Adiposidade	Direto	Pressão arterial	0,23	0,23
	Direto	Glicemia	0,16	0,16
CSE	Direto	Consumo de frutas e vegetais	0,47	0,4
Consumo de bebida alcóolica	Direto	Adiposidade	0,09	0,09
Consumo de frutas e vegetais	Direto	Pressão arterial	-0,11	-0,11
Controle médico da HAS	Direto	Pressão arterial	-0,09	-0,09

CSE: condição socioeconômica; HAS: hipertensão arterial sistêmica; AF: atividade física

## DISCUSSÃO

O presente estudo investigou as inter-relações entre PA, idade, sexo, condição socioeconômica, prática de AF, consumo de bebida alcoólica, tabagismo, consumo de frutas e vegetais, adiposidade e glicemia capilar por meio da MEE. Com a adoção dessa técnica multivariada, foi possível analisar um sistema composto por múltiplos fatores e visualizar suas inter-relações.

No modelo ajustado, foi identificado que a idade desempenha importante papel nesse sistema de inter-relações. Verificou-se efeito significativo da idade sobre PA, adiposidade, glicemia, consumo de frutas e vegetais, tabagismo, prática de AF e consumo de bebida alcóolica. Constatou-se que esse foi o fator que apresentou o maior efeito sobre a PA. Essa associação está bem descrita na literatura, na qual se relata tendência de aumento na prevalência de HAS com o avançar da idade^3 , 6 , 14^ , que traz mudanças intrínsecas ao processo de envelhecimento, como o enrijecimento das artérias e o aumento da resistência vascular periférica, o que tem repercussões na PA^14^ . O efeito indireto da idade sobre os níveis pressóricos, mediado principalmente pela adiposidade, foi também demonstrado neste estudo.

Verificou-se ainda que o aumento da idade se correlacionou com o aumento da adiposidade, corroborando resultado de outro estudo que observou aumento nas prevalências de obesidade corporal e obesidade central com o tempo^15^ . Nessa condição, são observadas modificações como redução de massa magra, aumento do percentual de gordura corpórea, diminuição da estatura, relaxamento da musculatura abdominal, alterações no IMC e na RCE^16^ . Entretanto, ressalta-se que, ao contrário do que ocorre em adultos, no idoso o peso diminui com a idade após atingir um platô em torno de 65 anos e 75 anos, respectivamente, nos homens e nas mulheres.

Este estudo demonstrou ainda que o aumento da idade esteve associado ao aumento dos níveis glicêmicos. Esse achado é refletido no aumento da prevalência de diabetes com o avançar da idade de uma população^17^ . Nesse sentido, a transição demográfica observada no Brasil nas últimas décadas tem sido apontada como uma das principais causas do aumento da incidência e prevalência do diabetes no país^18^ . Ressalta-se que hábitos e comportamentos não saudáveis como sedentarismo e alimentação inadequada também contribuem para alterações metabólicas^19^ ; entretanto, no presente estudo não foram evidenciados efeitos desses fatores nos níveis glicêmicos.

Foi constatado efeito da idade em comportamentos relacionados à saúde como tabagismo, consumo de frutas e vegetais, prática de AF e consumo de bebida alcoólica. Identificou-se efeito positivo da idade sobre o tabagismo, assim como observado em estudos anteriores^20^ , indicando que indivíduos com idade mais avançada consomem mais cigarros que os mais jovens. As campanhas de controle do tabagismo no Brasil, intensificadas a partir do final da década de 1980, articuladas pelo Ministério da Saúde, podem ser uma explicação para esse achado. Provavelmente o alcance dessas campanhas foi maior entre os indivíduos mais jovens, fazendo com que esses não adotassem o hábito tabagista, enquanto os mais velhos e fumantes persistem com essa prática.

O tabagismo tem sido considerado um forte preditor para o desenvolvimento de hipertensão arterial. Paradoxalmente a relação positiva entre níveis pressóricos e tabagismo não foi evidenciada no presente estudo. Assim, no modelo ajustado não ficou estabelecido que o aumento do número de cigarros consumidos esteja correlacionado com o aumento nos níveis pressóricos, independentemente da idade. Tal fato pode ser explicado pela natureza numérica e contínua em que a variável tabagismo foi medida (maços-ano), que provavelmente não possibilitou identificar seu efeito nos níveis pressóricos, uma vez que cerca de 77% dos indivíduos investigados declararam nunca ter fumado (maço-ano = 0). Outro aspecto a ser considerado se refere à tendência da normalização dos níveis pressóricos aproximadamente uma hora após o consumo do cigarro, o que pode ter ocorrido com os tabagistas da amostra.

Em relação à prática de AF, a idade apresentou pequeno efeito negativo, sugerindo que o aumento da idade está associado com a redução do tempo de prática semanal. Em parte, essa correlação pode decorrer de desigualdade no acesso, oferta e uso de recursos, equipamentos e serviços para a prática de AF, visto que esse comportamento pode ser influenciado por características ambientais e individuais. Um estudo^21^ realizado no município de São Paulo identificou decréscimo na frequência da prática de AF de acordo com a progressão da idade. Porém, entre os indivíduos de 50 a 59 anos, a frequência foi maior do que entre aqueles com idade de 30 a 39 e de 40 a 49 anos. Provavelmente, os indivíduos na faixa etária dos 50 aos 59 anos têm mais oportunidade para praticar AF ou necessitam realizá-la para promoção da saúde, com o objetivo de minimizar a progressão de agravos crônicos comuns no processo de envelhecimento^22^ .

Observou-se a mesma tendência no efeito da idade sobre o consumo de bebida alcóolica, ou seja, o avançar da idade mostrou-se correlacionado com menor consumo de bebida. Apesar de o efeito ter sido pequeno, este resultado corrobora os achados de estudo prévio^23^ . Nesse sentido, há uma tendência de maior consumo de álcool entre os jovens, o que, segundo a OMS^24^ , reflete um padrão mundial. Nesse período da vida, as reuniões em grupo se intensificam e o consumo de bebida alcoólica tende a aumentar, podendo chegar ao abuso. Assim, adolescentes e adultos jovens constituem a população de maior risco para consumo de álcool^25^ . Em contrapartida, um estudo prévio^21^ observou aumento no consumo de bebida alcóolica com a progressão da idade, fato que pode ser reportado como resultado do aumento na expectativa de vida e aumento da renda da população. Ressalta-se, entretanto, que 66% dos indivíduos pesquisados neste estudo relataram não fazer uso de bebidas alcóolicas.

No modelo ajustado foi também constatado efeito positivo da adiposidade sobre a PA, conforme achados de estudos anteriores^3 , 5^ , que observaram associação entre aumento do IMC e prevalência de HAS. Segundo a Sociedade Brasileira de Hipertensão (SBH)^4^ , 75% dos homens e 65% das mulheres apresentam HAS diretamente atribuída ao excesso de peso. Os mecanismos pelos quais o aumento da massa corporal interfere na alteração dos níveis de PA ainda não são completamente elucidados. Entretanto, essa associação pode ser atribuída ao acúmulo de gordura intra-abdominal que contribui para uma maior atividade simpática, que por sua vez aumentaria a reabsorção do sódio, o que resulta em um aumento da resistência vascular periférica e, consequentemente, da PA. Ademais, a gordura intra-abdominal também propicia o aumento das citocinas pró-inflamatórias, que podem interferir na elevação dos valores pressóricos^26^ .

No presente estudo foi verificado efeito positivo da adiposidade sobre a glicemia capilar, apesar de o efeito ter sido de baixa magnitude, assim como observado em outros estudos^17^ . Essa associação pode ser explicada pelo processo de infiltração de gordura no fígado, que interfere no metabolismo hepático, aumentando a resistência insulínica. Além disso, o excesso de gordura e glicose circulantes aumenta a demanda de secreção de insulina pelo pâncreas, levando as células produtoras de insulina ao estresse de funcionamento e exaustão^19^ .

Os achados deste estudo identificaram ainda o efeito positivo do consumo de bebidas alcoólicas sobre a adiposidade. Embora o efeito tenha sido pequeno, foi consistente com o observado em outro estudo^27^ . A bebida alcoólica é altamente calórica e, adicionada à dieta habitual, pode contribuir para alterações metabólicas e hormonais, com repercussões na homeostase energética, que interfere no apetite e no ganho de peso^27^ .

Foi verificado também efeito negativo, porém de baixa magnitude, do consumo de frutas e vegetais sobre a PA. Constatou-se que um maior consumo de frutas e vegetais esteve associado a menores níveis pressóricos, assim como identificado em estudo prévio^5^ . O consumo adequado de frutas e vegetais favorece a diminuição da PA devido à alta concentração de minerais com potencial hipotensivo nesses alimentos, em especial magnésio e potássio, e também por seu baixo teor de gordura^5^ .

O modelo ajustado identificou que os indivíduos do sexo masculino apresentaram maiores níveis pressóricos, o que pode estar relacionado à exposição a um maior número de fatores de risco presentes nessa população, como baixo nível educacional, consumo de álcool e baixa procura por serviços de saúde^4^ . Os resultados apontaram também que os homens fizeram maior uso de bebida alcoólica e de cigarro comparados com as mulheres do estudo.

O modelo ajustado apontou que a melhor condição socioeconômica esteve associada com maior frequência de consumo de frutas e vegetais. Outro estudo^28^ também já havia identificado essa associação. De fato, determinantes sociais como a escolaridade estão relacionados com o acúmulo de conhecimentos, capaz de elevar os cuidados com a saúde e aumentar hábitos saudáveis. Nesse sentido, pessoas com maior nível educacional demonstram uma percepção mais crítica sobre sua saúde e tendem a adotar um comportamento mais cuidadoso com ela. Maior escolaridade possibilita também melhores condições de trabalho e renda, que influenciam o acesso a alimentos mais saudáveis.

O consumo de alimentos ricos em gordura e sódio pode influenciar na elevação dos níveis pressóricos^1 , 3^ ; no entanto, o presente estudo não contemplou a avaliação desses parâmetros, constituindo assim uma limitação. Outra limitação decorre da ausência de informações a respeito da história familiar, uma vez que estimativas de herdabilidade têm demonstrado que 15% a 60% da variação da PA podem ser atribuídos a fatores genéticos^29^ .

Outra limitação do presente estudo se refere à análise direta dos dados provenientes de uma amostra por conglomerado, sendo o setor censitário a unidade amostral primária e não o indivíduo. Esse tipo de amostra demanda análise estatística no modo *survey* , o que não foi realizado no ajuste do modelo. Ao fazer a análise direta, corre-se o risco de obter associações espúrias em função da superestimação da qualidade de ajuste propiciada pelo aumento artificial do número de graus de liberdade. Por isso, recomenda-se cautela na interpretação das associações identificadas. Outra limitação desse estudo trata-se de sua natureza transversal, com os níveis pressóricos e as demais variáveis utilizadas no modelo medidos no mesmo momento, não sendo possível determinar a sequência temporal dos eventos. Assim, embora o modelo ajustado tenha possibilitado levantar hipóteses importantes de inter-relações, ele não confirmou causalidade.

Estabelecida a relação entre PA, adiposidade, glicemia, idade, sexo e comportamentos relacionados à saúde, pode-se conjecturar que elevações nos níveis pressóricos poderão se tornar progressivamente mais prevalentes na ausência de mudanças no comportamento da população, uma vez que a idade é um fator não modificável. Portanto, diante da tendência de envelhecimento da população brasileira, tornam-se necessárias ações em nível individual e populacional direcionadas à promoção da saúde, como intervenções que estimulem a alimentação saudável, prática regular de AF e restrições ao consumo de álcool e tabagismo, o que, provavelmente, impactaria de forma importante na redução da prevalência de PA elevada.
